# Genome-wide organellar analyses from the hornwort *Leiosporoceros dussii* show low frequency of RNA editing

**DOI:** 10.1371/journal.pone.0200491

**Published:** 2018-08-08

**Authors:** Juan Carlos Villarreal A., Monique Turmel, Maurane Bourgouin-Couture, Jérôme Laroche, Noris Salazar Allen, Fay-Wei Li, Shifeng Cheng, Karen Renzaglia, Claude Lemieux

**Affiliations:** 1 Département de Biologie, Université Laval, Québec, Canada; 2 Institut de Biologie Intégrative et des Systèmes (IBIS), Université Laval, Québec, Canada; 3 Smithsonian Tropical Research Institute, Panama City, Panama; 4 Département de biochimie, de microbiologie et de bio-informatique, Université Laval, Québec, Canada; 5 Plant Biology Section, Cornell University, Ithaca, New York, United States of America; 6 BGI-Shenzhen, Beishan Industrial Zone, Shenzhen, China; 7 Department of Plant Biology, Southern Illinois University, Carbondale, Illinois, United States of America; Estacion Experimental del Zaidin - CSIC, SPAIN

## Abstract

Because hornworts occupy a pivotal position in early land colonization as sister to other bryophytes, sister to tracheophytes, or sister to all other land plants, a renewed interest has arisen in their phylogenetic diversity, morphology, and genomes. To date, only five organellar genome sequences are available for hornworts. We sequenced the plastome (155,956 bp) and mitogenome (212,153 bp) of the hornwort *Leiosporoceros dussii*, the sister taxon to all hornworts. The *Leiosporoceros* organellar genomes show conserved gene structure and order with respect to the other hornworts and other bryophytes. Additionally, using RNA-seq data we quantified the frequency of RNA-editing events (the canonical C-to-U and the reverse editing U-to-C) in both organellar genomes. In total, 109 sites were found in the plastome and 108 in the mitogenome, respectively. The proportion of edited sites corresponds to 0.06% of the plastome and 0.05% of the mitogenome (in reference to the total genome size), in contrast to 0.58% of edited sites in the plastome of *Anthoceros angustus* (161,162 bp). All edited sites in the plastome and 88 of 108 sites in the mitogenome are C-to-U conversions. Twenty reverse edited sites (U-to-C conversions) were found in the mitogenome (17.8%) and none in the plastome. The low frequency of RNA editing in *Leiosporoceros*, which is nearly 88% less than in the plastome of *Anthoceros* and the mitogenome of *Nothoceros*, indicates that the frequency of RNA editing has fluctuated during hornwort diversification. Hornworts are a pivotal land plant group to unravel the genomic implications of RNA editing and its maintenance despite the evident evolutionary disadvantages.

## Introduction

Bryophytes—liverworts, mosses and hornworts—have in common a haploid-dominant life cycle. Because of their pivotal evolutionary position, bryophytes have been targets of several phylogenomic analyses to disentangle their interrelationships, resulting in three main competing and equally supported hypotheses. The liverwort-sister hypothesis suggests that bryophytes are paraphyletic with liverworts sister to all other land plants and hornworts sister to tracheophytes [1]. The hornwort-sister hypothesis supports hornworts as sister to all other land plants [2,3,4]. The third hypothesis identifies bryophytes as monophyletic with hornworts sister to a moss plus liverwort clade [3, 4].

From this debate, it has become evident that hornworts have a crucial place in the evolution of early land plants, prompting a renewed interest in their phylogenetic diversity, morphology, and genomes [5,6]. Three recently launched nuclear genome projects are targeting two species within the genus *Anthoceros* (*A*. *agrestis*, *A*. *punctatus*) and the monotypic *Leiosporoceros* (*L*. *dussii*), the sister taxon to all hornworts [1,7] (OneKP unpublished data). The *Leiosporoceros* genome project targets *L*. *dussii* due to its crucial phylogenetic position, small genome size (approximately 160 Mbp) and its morphological innovations that include a unique symbiotic arrangement of endophytic cyanobacteria, isobilateral tetrad development and spore architecture [5,7].

To date, only two chloroplast (plastome) and three mitochondrial (mitogenome) genome sequences are available for hornworts. The plastomes of *Anthoceros angustus* and *Nothoceros aenigmaticus* are collinear and only differ by the expansion of the inverted repeat and the presence of an intron in the large subunit rRNA gene in *A*. *angustus* [8,9]. The mitogenomes of *A*. *angustus*, *Phaeoceros laevis* and *N*. *aenigmaticus* show more differences, including four inversions, 7–10 genes in different stages of pseudogenization and additional introns in *P*. *laevis* and *A*. *angustus* [10,11,12]. The near collinearity of hornwort organellar genomes is hardly surprising considering the organellar genomes of liverworts, hornworts and mosses have maintained a conserved gene order and content despite millions of years of separate evolutionary histories.

The most important characteristic found in organellar genomes of hornworts is the high level of RNA editing relative to other bryophytes [13,14,15]. RNA editing is a form of nucleotide sequence alterations that occurs at the transcription level [13,14]. RNA editing converts cytidines to uridines (C-to-U or canonical RNA editing) or uridines to cytidines (U-to-C or so-called reverse editing) in the primary transcript prior to translation [14]. This converts a sense codon into a more evolutionary conserved one or a start/stop codon to a sense codon. However, RNA editing is not only restricted to coding regions; it also occurs in introns and untranslated regions (UTRs) but is rare in rRNA transcripts [14]. RNA editing in introns and tRNAs allows the proper folding and processing of the molecules involved [14,16]. The widespread occurrence of the canonical RNA editing contrasts sharply with the restricted phylogenetic distribution of reverse editing, which among land pants has been reported only for hornworts, lycophytes and ferns [14]. This restricted distribution raises questions as to whether reverse editing evolved in the most recent common ancestor of hornworts + tracheophytes or independently in hornworts and early tracheophytes.

The *A*. *angustus* plastome is the only hornwort organellar genome for which complete cDNA sequences are known. It shows an elevated rate of RNA editing with up to 942 sites, including 509 C-to-U and 433 U-to-C conversions. This is the highest frequency of reverse editing among land plants [8,15,17]. In contrast, preliminary analyses of targeted plastid and mitochondrial genes in 12 hornwort taxa suggested that no or little RNA editing occurs in *L*. *dussii* [18,19]. Analyses of specific regions of the plastid *rbcL*, *rpoC*, and *atpB* genes revealed no edited sites in *Leiosporoceros* [19]. In the examined 1,228-bp region of *rbcL* alone, a total of 72 edited sites (43 C-to-U and 28 U-to-C conversions) were identified in the other taxa, with 20–34 edited sites per taxon. The mitochondrial *nad5* gene of *Leiosporoceros* displayed only 0.7% of edited sites (8/110), while edited bases accounted for 3.1–4.1% of the sites in other hornworts [18]. A partial cDNA analysis of the *N*. *aenigmaticus* mitogenome revealed the presence of 422 edited sites [11]. These studies therefore suggest that RNA editing of organelle transcripts are quite variable across the different hornwort lineages.

The availability of only a few organellar transcriptomes from early land plants precludes genome-wide analyses of RNA editing, thus hampering the reconstruction of a clear evolutionary trend for this widespread phenomenon. The complete absence of RNA editing in complex thalloid (marchantioids) liverworts is puzzling [20] and it may be related to the low substitution rate of organellar genes in this clade [21]. In mosses, RNA editing occurs at very low frequency in organellar genomes, with 2–11 sites of the flagship species *Physcomitrella patens* [22] or higher levels in single gene analysis in *Takakia*. Among the seed-free tracheophytes ferns and lycophytes, there exist high heterogeneity in the levels of RNA editing [23]. In contrast, early branching lineages of angiosperms seem to display frequent RNA editing [24].

Given the critical phylogenetic position of *L*. *dussii* within hornworts, we sequenced and assembled the plastome and mitogenome of this species. Using high-coverage transcriptome sequencing, we also studied the extent of RNA editing in both organelles of *L*. *dussii* to assess whether the apparent lack of editing in plastids and the low rate of editing in the mitochondrial *nad*5 gene are organellar-wide phenomena. Our study was thus aimed at elucidating critical information in reconstructing organelle genome evolution in early land plants.

## Material and methods

### Sample acquisition

Material of *Leiosporoceros dussii* (Steph.) Hässel (Villarreal, PA-15-1479) was collected in Río el Guayabo, El Valle de Antón, Prov. De Coclé, Panamá and brought to the laboratory for immediate DNA extraction.

### Organellar genome sequencing and annotation

Two separate genomic total DNA preparations were obtained for *L*. *dussii* using the Power Plant@ ProDNA isolation kit (MoBIo). The preparations were used to produce sequencing libraries with the Nextera DNA Sample Prep Kit (Illumina, San Diego, CA, USA). Sequencing of the two libraries was performed at the Smithsonian Tropical Research Institute (STRI, Panama), using Illumina MiSeq technology. Over 9 million paired-end reads were obtained for the first library and 8 million paired-end reads for the second library. Low-quality reads were trimmed using CLC Genomics Workbench (CLC Bio, Aarhus, Denmark) and *de novo* assemblies were conducted using A5 in CLC Genomics Workbench (CLC Bio, Aarhus, Denmark). Contigs of the plastome and mitogenome were identified by BlastN similarity searches (E-value < 10−5) against the *A*. *angustus* plastome (NC_004543.1) and *P*. *laevis* mitogenome (GQ376531.1), respectively. Final assemblies were completed using Sequencer 5.1 (Gene Codes Corporation, Ann Arbor, Michigan, USA).

To annotate the two organelle genomes, we used a custom-built suite of bioinformatics tools allowing the automated execution of the following three steps: (1) ORFs were found using GETORF in EMBOSS [25], (2) their translated products were identified by BlastP searches [26] against a local database of plastome- or mitogenome-encoded proteins or the nr database at the National Center for Biotechnology Information, and (3) consecutive 100-bp segments of the genome sequence were analyzed with BlastN and BlastX to determine the approximate positions of coding genes, introns and exons. The precise positions of rRNA and tRNA genes were identified using RNAmmer [27] and tRNAscan-SE [28], respectively. Intron boundaries were determined by manual modeling of intron secondary structures [29,30] and by comparing the sequences of intron-containing genes with those of intronless homologs. Circular genome maps were drawn with OGDraw [31].

### Transcriptome sequencing and analysis of RNA editing

RNA was recovered from the same colony that was used for DNA sequencing. Separate samples of this colony were placed in RNA Later (Ambion, Life Technologies, CA, USA) and shipped to the Beijing Genomic Institute in Shenzhen (BGI-Shenzen) where a rRNA-depleted library was constructed using the Ribo-Zero Plant Leaf rRNA Removal Kit (Epicentre, city, country) using random hexamers. A transcriptome library was constructed using a TruSeq mRNA stranded sample preparation kit (Illumina, Inc.). Sequencing of this library was performed using a HiSeq instrument, which yielded a total of 45,351,761 reads (over 9 billion bp).

Illumina RNA-seq data were mapped to both organellar genomes using CONSED [32]. Within CONSED, we used a custom PERL script « addSolexaReads » that uses a searching script called « Crossmatch » [32] (developed by David Gordon and Phil Green, U. of Washington). Within Crossmatch we used the following settings: -discrep_lists -tags -masklevel 0 -minscore 25 -gap1_only -repeat_screen 2. After mapping and filtering each genome, the coverage of the RNA reads was aligned and scrutinized manually using Geneious 9.0.5 (Biomatters Limited) to identify sites of RNA editing in coding regions, introns and intergenic spacers. The editing efficiency of each site was estimated by determining the proportion of cDNA reads that contained the edited nucleotide. All edited sites have been identified at least in 12% of the reads in the plastome and 5% in the mitogenome (ranging from 165–65,770 reads per site in the plastome and 102–44,015 in the mitogenome, S3 and S4 Figs). Edited sites in protein-coding genes were classified based on whether they remained similar (conservative sites), changed (non-conservative sites), or did not alter amino acid conservation (synonymous or silent sites) relative to orthologous proteins from green algae and other land plants. For this classification, multiple protein alignments were carried out using Muscle 3.5 [33]. The RNA edited sites in *Leiosporoceros* were compared to those previously reported for the organelles of other hornworts [10,11,15, 34]. The recently released cDNA analysis of the mitogenome of *Nothoceros aenigmaticus* was used for comparison. We avoided comparisons with the mitogenome of *P*. *laevis* because the analysis was done using an RNA-editing predicting software [34].

## Results and discussion

### Plastome organization

**Fig 1:**
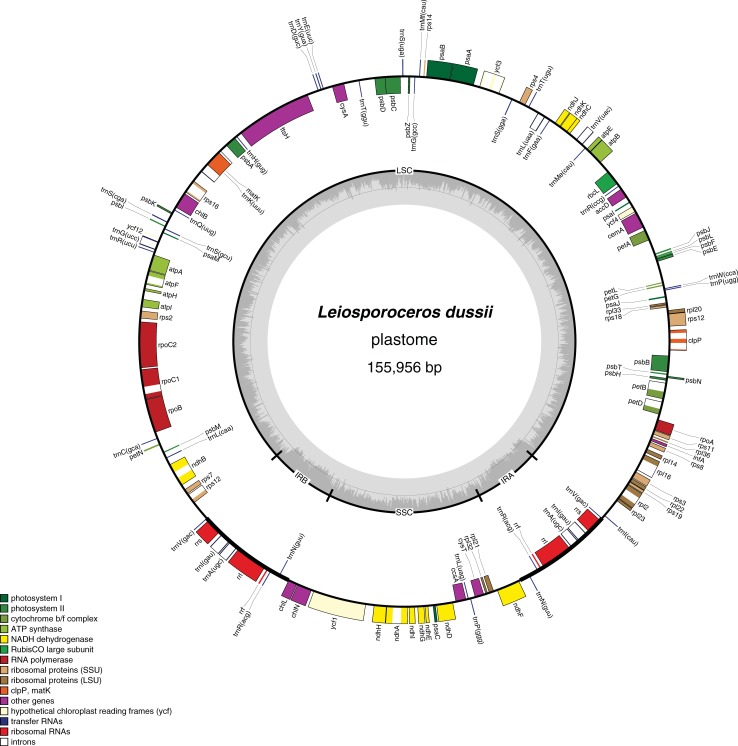
Map of the *L*. *dussii* plastome. Genes (exons are denoted as closed boxes) on the outside of the outermost circle are transcribed in the counter clockwise direction, while genes on the inside of this circle are transcribed in the clockwise direction. Structural components are labeled on the inner circle as LSC and SSC regions, IR_A_ and IR_B_. Inner graph charts % GC content across the genome. A color-coded scale classifies the genes into functional categories.

At 155,956 bp the circular *Leiosporoceros* plastome is about the same size as *Anthoceros* (161,162 bp) and *Nothoceros* (153,208 bp) plastomes (Table 1; Fig 1; Genbank accession MH577299). It features two identical inverted repeat (IR) regions of 9,693 bp, a large single-copy (LSC) region of 114,140 bp, and a small single-copy (SSC) region of 22,432 bp. The GC content of the entire plastome is 30.9%, whereas that of the IR alone is 46.5% due to elevated GC content of rRNA genes. The *Leiosporoceros* plastome contains 85 protein-coding genes, 4 rRNA genes, and 32 tRNA genes. Although the overall structure and gene order are identical to those of *Anthoceros* and *Nothoceros* [8,9], it differs at the IRa/LSC boundary, content of genes and pseudogenes, and intron content (Table 1, Fig 1). Unlike the *Anthoceros* plastome and similar to that of *Nothoceros*, the *ndhB* gene, *rps7* and the 3’ *rps12* exon are not included in the IR; instead these loci occur in the LSC region. Furthermore, a ~684-bp group I intron that is inserted within the large subunit rRNA gene (*rrl*) of *Anthoceros* and a number of green algae is absent from the IRs of both *Leiosporoceros* and *Nothoceros*, as well as all other hornworts and other land plants. The absence of this *rrl* intron in all hornworts examined thus far except *Anthoceros*, points to a potential horizontal transfer between a green alga and species of *Anthoceros* [9]. Additional differences are the presence of *trnS* (CGA) and *matK* in *L*. *dussii*, which are absent or occur as pseudogenes in *A*. *angustus* and *N*. *aenigmaticus* (Table 1).

**Table 1:** Summary table of the genes present in hornwort plastomes, + gene presence, ψ pseudogene.

Genes	*Leiosporoceros dussii*	*Anthoceros angustus*	*Nothoceros aenigmaticus*
**Proteins not related to photosynthesis**			
accD	+	+	+
cysA	+	+	+
cysT	+	+	+
**ATP synthase**			
atpA	+	+	+
atpB	+	+	+
atpE	+	+	+
atpF	+	+	+
atpH	+	+	+
atpI	+	+	+
**Chlorophyll photosynthesis**			
chlB	+	+	+
chlL	+	+	+
chlN	+	+	+
clpP	+	+	+
**Translation factor**			
infA	+	+	+
**Miscellaneous proteins**			
ycf1	+	+	+
ftsH (ycf2)	+	+	+
ycf3	+	+	+
ycf4	+	+	+
matK	+	*ψ*	*ψ*
ccsA	+	+	+
**NADH dehydrogenase**			
ndhA	+	+	+
ndhB	+	+	+
ndhC	+	+	+
ndhD	+	+	+
ndhE	+	+	+
ndhF	+	+	+
ndhG	+	+	+
ndhH	+	+	+
ndhI	+	+	+
ndhJ	+	+	+
ndhK	+	+	+
**Cytochrome**			
petA	+	+	+
petB	+	+	+
petD	+	+	+
petG	+	+	+
petL	+	+	+
petN	+	+	+
**Photosystem I**			
psaA	+	+	+
psaB	+	+	+
psaC	+	+	+
psaI	+	+	+
psaJ	+	+	+
psaM	+	+	+
psbA	+	+	+
psbB	+	+	+
psbC	+	+	+
psbD	+	+	+
psbE	+	+	+
psbF	+	+	+
psbH	+	+	+
psbI	+	+	+
psbJ	+	+	+
psbK	+	+	+
psbL	+	+	+
psbM	+	+	+
psbN	+	+	+
psbT	+	+	+
psbZ	+	+	+
**Rubisco**			
rbcL	+	+	+
**Ribosomal proteins–Large subunits**			
rpl2	+	+	-
rpl14	+	+	+
rpl16	+	+	+
rpl20	+	+	+
rpl21	+	+	+
rpl22	+	+	+
rpl23	+	+	+
rpl32	+	+	+
rpl33	+	+	+
rpl36	+	+	+
**Ribosomal proteins–Small subunits**			
rps2	+	+	+
rps3	+	+	+
rps4	+	+	+
rps7	+	+	+
rps8	+	+	+
rps11	+	+	+
rps12	+	+	+
rps14	+	+	+
rps15	-	*ψ*	-
rps16	+	+	+
rps18	+	+	+
rps19	+	+	+
**Translation / translation RNA polymerase**			
rpoA	+	+	+
rpoB	+	+	+
rpoC1	+	+	+
rpoC2	+	+	+
**Ribosomal proteins**			
23S	+	+	+
16S	+	+	+
5S	+	+	+
4.5S	+	+	+
**Transfer RNAs**			
trnA(ugc)	+	+	+
trnC(gca)	+	+	+
trnD(guc)	+	+	+
trnE(uuc)	+	+	+
trnF(gaa)	+	+	+
trnG(gcc)	+	+	+
trnG(ucc)	+	+	+
trnH(gug)	+	+	+
trnI(cau)	+	+	+
trnI(gau)	+	+	+
trnK(uuu)	+	+	+
trnL(caa)	+	+	+
trnL(uaa)	+	+	+
trnL(uag)	+	+	+
trnMe(cau)	+	+	+
trnMf(cau)	+	+	+
trnN(guu)	+	+	+
trnP(ggg)	+	+	+
trnQ(uug)	+	+	+
trnR(acg)	+	+	+
trnR(ccg)	+	+	+
trnR(ucu)	+	+	+
trnS(cga)	+	-	-
trnS(gcu)	+	+	+
trnS(gga)	+	+	+
trnS(uga)	+	+	+
trnT(ggu)	+	+	+
trnT(ugu)	+	+	+
trnV(gac)	+	+	+
trnV(uac)	+	+	+
trnW(cca)	+	+	+
trnY(gua)	+	+	+

### Mitogenome organization

**Fig 2:**
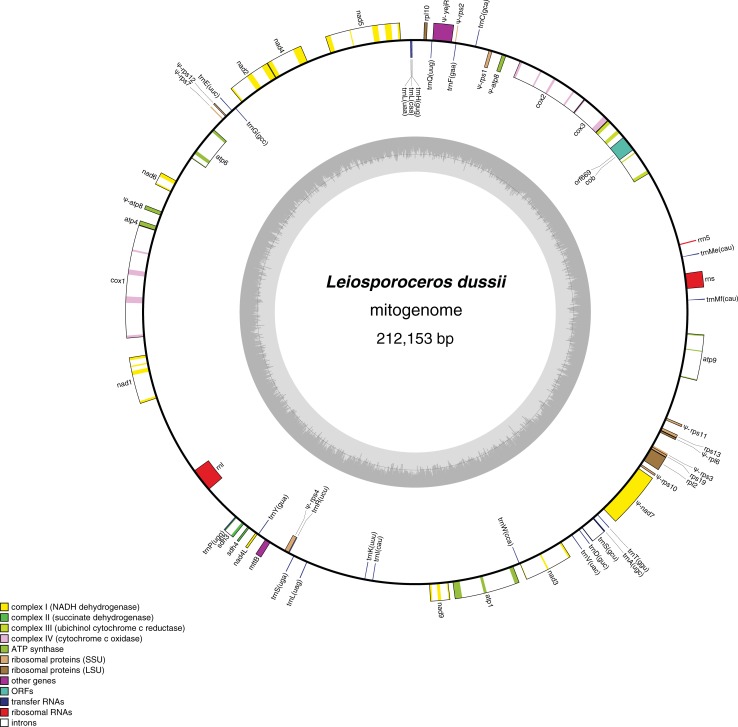
Map of the *L*. *dussii* mitogenome. Genes (exons indicated as closed boxes) on the outside of the circle are transcribed in the clockwise direction, and genes on the inside of the circle are transcribed in the counter clockwise direction. Pseudogenes are marked with a ψ. A color-coded scale classifies the genes into functional categories.

The circular *Leiosporoceros* mitogenome is 212,153 bp long (Table 2; Fig 2; Genbank accession MH577300) and its GC content is 44.2%. It contains 32 protein-coding genes (including pseudogenes), 3 rRNA genes, and 23 tRNA genes. The *Leiosporoceros* mitogenome is larger than those of *P*. *laevis* (209,482 bp) and *N*. *aenigmaticus* (184,908 bp) [10, 11], but smaller than *A*. *angustus* (242,410 bp). It also has the highest number of functional genes, including three protein-coding genes (*rpl2*, *rps19*, *sdh3*) and three tRNA genes (*trnS* (UCU), *trnS* (UGA), *trnS* (GCU) that are either absent or pseudogenized in *P*. *laevis* and *N*. *aenigmaticus* (Table 2). In total, there are twelve pseudogenes in *Leiosporoceros*, none of which correspond to a functional gene in the *N*. *aenigmaticus* and *P*. *laevis* (Table 2).

**Table 2:** Summary table of the genes present in hornwort mitogenomes, + gene presence, ψ pseudogene.

Genes	*Leiosporoceros dussii*	*Nothoceros aenigmaticus*	*Phaeoceros laevis*	*Anthoceros angustus*
**Respiratory chain complex V**				
atp1	*+*	*+*	*+*	*+*
atp4	*+*	*+*	*+*	*+*
atp6	*+*	*+*	*+*	*+*
atp8	*Ψ*	*ψ*	*ψ*	*+*
atp9	*+*	*+*	*+*	*+*
Cythochrome c biosis				
ccmFC (yejR)	*ψ*	*ψ*	*ψ*	*ψ*
**Respiratory chain complex III**				
cob a	*+*	*+*	*+*	*+*
**Respiratory chain complex IV**				
cox1	*+*	*+*	*+*	*+*
cox2	*+*	*+*	*+*	*+*
cox3	*+*	*+*	*+*	*+*
**Respiratory chain complex I**				
nad1	*+*	*+*	*+*	*+*
nad2	*+*	*+*	*+*	*+*
nad3	*+*	*+*	*+*	*+*
nad4	*+*	*+*	*+*	*+*
nad4L	*+*	*+*	*+*	*+*
nad5	*+*	*+*	*+*	*+*
nad6	*+*	*+*	*+*	*+*
nad7	*ψ*	*ψ*	*ψ*	*ψ*
nad9	*+*	*+*	*+*	*+*
**Ribosomal proteins**				
Rpl2	*+*	*-*	*ψ*	*-*
Rpl5	*-*	*ψ*	*-*	*-*
rpl6	*ψ*	*ψ*	*ψ*	*ψ*
Rpl10	*+*	*+*	*+*	*+*
rps1	*ψ*	*ψ*	*v*	*ψ*
rps2	*ψ*	*-*	*ψ*	*ψ*
rps3	*ψ*	*-*	*-*	*-*
rps4	*ψ*	*ψ*	*ψ*	*ψ*
rps7	*ψ*	*ψ*	*ψ*	*ψ*
rps8	*-*	*ψ*	*-*	*-*
rps10	*ψ*	*-*	*-*	*-*
rps11	*ψ*	*ψ*	*ψ*	*ψ*
rps12	*ψ*	*ψ*	*ψ*	*ψ*
rps13	*+*	*+*	*+*	*-*
rps14	*-*	*+*	*-*	*-*
rps19	*+*	*-*	*-*	*-*
**Respiratory chain complex II**				
sdh3	*+*	*ψ*	*ψ*	*ψ*
sdh4	*+*	*+*	*+*	*+*
**Other proteins**				
tatC	*+*	*+*	*+*	*+*
**Ribosomal RNA**				
rrn18	*+*	*+*	*+*	*+*
rrn26	*+*	*+*	*+*	*+*
rrn5	*+*	*+*	*+*	*+*
rrn 4.5S				
**Transfer RNAs**				
trnA(UGC)	*+*	*+*	*+*	*+*
trnC(GCA)	*+*	*+*	*+*	*+*
trnD(GUC)	*+*	*+*	*+*	*+*
trnE(UUC)	*+*	*+*	*+*	*+*
trnF(GAA)	*+*	*+*	*+*	*+*
trnG(GCC)	*+*	*+*	*+*	*+*
trnH(GUG)	*+*	*+*	*+*	*+*
trnI(CAU) b	*+*	*+*	*+*	*+*
trnK((UUU)	*+*	*+*	*+*	*+*
trnL(CAA)	*+*	*+*	*+*	*+*
trnL(UAA)	*+*	*+*	*+*	*+*
trnL(UAG)	*+*	*-*	*+*	*+*
trnM(CAU)	*+*	*+*	*+*	*+*
trnM*f(CAU)*	*+*	*+*	*+*	*+*
trnP(UGG)	*+*	*+*	*+*	*+*
trnQ(UUG)	*+*	*+*	*+*	*+*
trnR(UCU)	*+*	*-*	*-*	*+*
trnS(UGA)	*+*	*-*	*-*	*+*
trnS(GCU)	*+*	*-*	*-*	
trnT(GGU)	*+*	*+*	*+*	*ψ*
trnV(UAC)	*+*	*-*	*+*	*+*
trnW(CCA)	*+*	*+*	*+*	*+*
trnY(GUA) c	*+*	*+*	*ψ +*	*+*

Footnotes

a There is an ORF in the second intron (orf 669)

b C in the first position of the anticodon assumed to be post-transcriptionally modified to lysidine, which pairs with A rather than G

c The trnYgua in *Phaeoceros* mtDNA has two copies, with one pseudogenized. There are also two copies of atp8, both are pseudogenes.

The *Leiosporoceros* mitogenome also contrasts with its *P*. *laevis* and *N*. *aenigmaticus* relatives at the intron content level. All of the 35 introns are *cis*-spliced and with the exception of the first intron in the *cob* gene, belong to the group II class. Only the first (group I) and second introns located in *cob*, feature an open reading frame. Twenty-six of the *Leiosporoceros* group II introns are present at identical positions in both the *P*. *laevis* and *N*. *aenigmaticus* mitogenomes, and three additional ones are shared with *P*. *laevis* alone. Of these 29 hornwort introns, a small fraction is shared with other bryophytes and/or vascular plants. These results indicate that most of the introns found in hornworts were acquired early during their evolutionary history and were transmitted by vertical descent.

### RNA editing in plastids and mitochondria

RNA editing events were investigated in every transcript derived from the *Leiosporoceros* plastome and mitogenome (S1 Fig, Tables 2 and 3, S1 Table). The RNA sequence data were mapped on both organellar genomes and the coverage of the RNA reads was scrutinized manually. The proportion of edited sites corresponds to 0.06% of the plastome and 0.05% of the mitogenome (in reference to the total genome size). In total, 109 sites were found in the plastome and 108 in the mitogenome, respectively (Table 1, S1 and S2 Tables). All edited sites in the plastome are C-to-U conversions, contrasting with 88 sites in the mitogenome. Nineteen reverse edited sites (U-to-C conversions) were found in the mitogenome (17.8%) and none in the plastome. We identified 26 putative base conversions in the rRNA, half of which are unusual conversions (G→T, A→G, T→A and G→A); however, BlastN analysis of the reads containing the divergent nucleotides revealed that most, if not all of them, originated from RNA contaminants derived mostly from vascular plants. We also found four types of polymorphisms (A→G, T→G and G→T), representing 33% to 88% of all reads, in the *clpP* (A→G) and *psaA* (T→G) genes and in three intergenic spacers (*ycf4*-*psaI*, *trnD*(guc)-*ftsH* and *atpH*-*atpI*) of the plastome (S1 Table). BlastN analyses of the reads revealed no match to any hornworts or other land plants, suggesting contamination or sequencing errors. In the mitogenome, such sites were identified in four introns and two intergenic spacers (*trnl*(uaa)-*nad5* and *mttB*-*rps4*), and accounted for 24 to 86% of all reads (S2 Table). The latter sites were further excluded from analyses and were attributed to either DNA polymorphisms that arose when mixing several gametophytes during DNA extraction [18] or sequencing errors. Further studies are being conducted in our research groups to further dissect the nature of such anomalous editing variants.

**Table 3:** Nature and density of RNA edited sites in the organellar genomes of *Leiosporoceros* and other hornworts.

Plastome						Mitogenome			
Genome location	Editing event	*L*. *dussii*		*A*. *angustus*		*L*. *dussii*		*N*. *aenigmaticus*	
			%		%		%		%
**Total**	** **	**109**		939		**108**		422	
	**C→ U**	109	100	507	54.0	88	82.2	361	82,9
	**U→ C**	0	0	432	46.0	20	17,7	61	17,1
**Protein-coding genes**	** **	**102**				**94**			
** **	**1st codon position**	5	4.9	371	39.8	29	30.1	124	-
** **	**2nd position**	96	94.11	532	57.1	57	61.2	247	-
** **	**3rd position**	1	0.99	28	3.0	8	8.62	21	-
** **	**Start codon created**	1		5	2.9	2	2,1	-	-
** **	**Stop codon created**	1		0	0	0	0	-	-
** **	**Stop removed**	0	0	164	97.0	9	-	-	-
**tRNA and rRNA genes**	** **	0		1		**1**		-	-
	**tRNA**	0	0	1	1	1	0.92	-	-
	**rRNA**	0	0	0	0	0	0	-	-
**Non-coding regions **		7							
	**Intron**	2	1.90	3	37.5	2	1.85	-	-
	**UTRs**	5	7.61	4	50.0	10	9.25	-	-

Data from *N*. *aenigmaticus* have been submitted to Genbank and await further processing [12,16]. One edited site in the *nad7* pseudogene is included. Percentages are estimated excluding this site. One site is found in the orf 669, the orf is located within the the 3^rd^ intron of cob.

**Fig 3:**
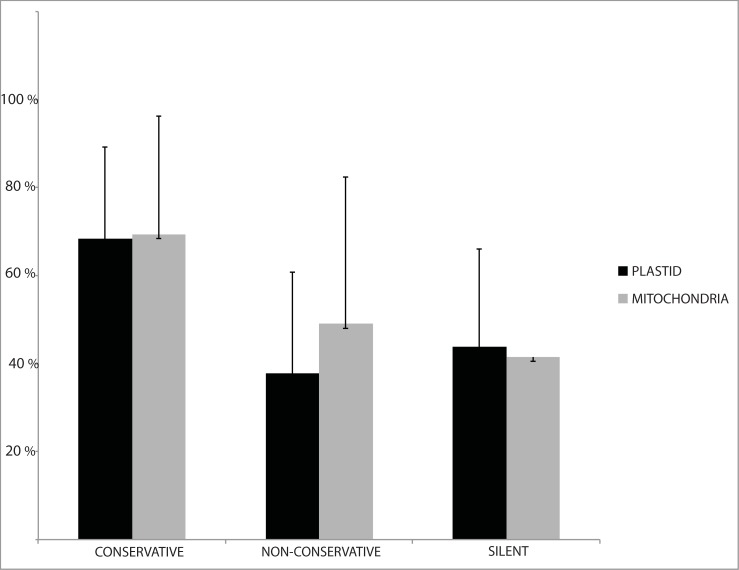
Functional consequence and efficiency of RNA editing in the protein-coding regions of *L*. *dussii* plastid and mitochondrial transcripts. The edited sites were classified into three categories: conservative (when the editing events improved sequence conservation to orthologous proteins from green algae or other land plants), non-conservative (when they reduced sequence conservation), and synonymous or silent sites (when the amino acids corresponding to these sites were not altered).

The majority of RNA edited sites in the plastome (102/109) and mitogenome (94/108) affect coding regions of protein-coding genes, most frequently the second codon position (Table 3). In the mitogenome, U-to-C conversions (17 in coding regions) were found at first (9 sites) and second (8) codon positions. As expected, synonymous edited sites are mostly found at third positions and appear to be randomly distributed in both organelle genomes. The majority of conservative edited sites show the highest efficiency of editing in the plastome (68.3%) and the mitogenome (69.4%). The silent edited sites have a lower efficiency of editing (32.4% in the plastome and 41.4% in the mitogenome) than conservative and non-conservative sites but still have a high score (Fig 3).

Of the 102 codons edited in the plastome, 57% are serine codons: 43 are changed into leucine and 16 into phenylalanine codons (Table 4). The second most important editing event (23/102) occurs in proline codons, which are all changed into leucine codons. Similarly, out of the 93 edited codons in the mitogenome, serine codons are the most frequently edited (26/93), leading to the conversion of this amino acid to leucine, phenylalanine, or methionine (Table 4). Moreover, as observed for the plastome, proline codons are the second most frequently edited codons (17/93), leading to leucine and serine. These observations are entirely consistent with (at a lower scale) the editing pattern reported for the *A*. *angustus* plastome, where serine codons are the most altered and are mainly converted to leucine and phenylalanine codons [15].

**Table 4:** Conversion of amino acids resulting from RNA editing in the plastome and mitogenome of *L*. *dussii*.

	Plastome		Mitogenome	
Amino acid	Number	Conversion	Number	Conversion
Thr	10	5 Ile, 5 Met (1 start),	7	5 Met (2 start codons), 2 Ile
Gln	1	Stop		
Pro	23	Leu	17	1 Ser, 16 Leu
Arg	2	Cys	13	4 Trp, 9 Cys
Leu	3	1Leu, 2Phe	9	1 Phe, 2 Ser, 2 Pro, 4 Leu
Ala	4	2 Val, 2 Ala		
Gly				
Ser	59	43 Leu, 16 Phe	26	1 Met, 12 Phe, 13 Leu
His			4	4 Tyr
Val			1	1 Ala
Ile			2	Ile, Ser
Phe			6	2 Ser, 4 Phe
Stop codon			9	3 Gln, 6 Arg

However, despite this similarity, the *Leiosporoceros* and *Anthoceros* plastomes show striking differences in RNA editing frequency with 109 edited sites in *Leiosporoceros* versus 939 sites in *Anthoceros* [15] (Table 2). In addition, the absence of reverse editing in *Leiosporoceros* contrasts sharply with the abundance of U-to-C conversions (432) observed in *Anthoceros*. Re-establishment of proper start codons and removal of stop codons are more important in *A*. *angustus* (164 codons removed) compared to *L*. *dussii* (only one start codon and one stop codon created and no stop codons removed). This may explain the lower number of edited sites in *L*. *dussii*, in particular the absence of U-to-C editing. Only 37 edited sites are shared between the *L*. *dussii* and *Anthoceros* plastomes (S2 Fig), suggesting lineage-specific editing rather than a conservation of edited sites across the hornwort phylogeny. More than half of the non-synonymous changes observed in *Leiosporoceros* correspond to codons unaffected by RNA editing in *Anthoceros*.

Surprisingly, the number of edited sites is similar in the *L*. *dussii* plastome (109) and mitogenome (108), an unusual case among land plants. The number of edited sites in the mitogenome is higher in *Nothoceros* than in *Leiosporoceros* (over 422 vs 108), with much fewer U-to-C conversions in *Leiosporoceros* than in *Nothoceros* (19 sites vs 61) (Table 2) and a low proportion of shared edited sites between these two hornwort mitogenomes.

Analysis of the plastid gene *rbcL* across the hornwort phylogeny showed up to 72 edited sites, 20 of which were present in *A*. *angustus* and 34 in *Phaeomegaceros coriaceus* [19]. Nearly 61% of the edited sites were of the canonical C-to-U type and the remainder the U-to-C type [18]. Similarly, the mitochondrial *nad*5 gene was heavily edited with up to 45 edited sites in *Nothoceros fuegiensis* [19], out of a total of 125 sites across the hornwort phylogeny. Nearly 68% of the edited sites were of the canonical C-to-U type and the remaining were U-to-C type [18]. If this partial RNA editing analysis represents a genome-wide phenomenon, then we predict that the evolutionary pathways of RNA editing in hornworts varies across different lineages, invoking the need of further hornwort and land plant organellar genomes to elucidate patterns of RNA editing evolution [24].

Elucidating the mechanisms of RNA editing across land plants, in particular the origin and maintenance of reverse editing, remains an active field of research. Regarding the canonical RNA editing, a vast amount of research has detailed the role of the editosome and the pentatricoptide repeat proteins (PPR) [14]. A recent preliminary survey of the genome of the hornwort *A*. *agrestis* identified the PPR domains as the most abundant repeat types in the nuclear genome [6]. The large number of predicted edited sites in the organellar genomes of *Anthoceros* is hand-to-hand with the proliferation of the PPR domains [6]. But at this point, cDNA data from the plastome of *A*. *angustus* is the only reliable data from a hornwort organelle to compare with the transcriptomic data we generated for *Leiosporoceros*. *Leiosporoceros* represents an ideal candidate to study the expansion of the editosome across hornworts and land plants. The reduced number of edited sites could facilitate the search for PPR domains and related proteins involved in the organellar editosomes.

## Conclusions

We sequenced both organellar genomes of a crucial lineage in the hornwort phylogeny [4,7] and present the first fully sequenced transcriptome derived from a hornwort mitogenome. The low frequency of RNA editing in *Leiosporoceros* with nearly 88% less in the plastome than *Anthoceros* suggests idiosyncratic and highly variable levels of RNA editing during hornwort diversification, advocating for the need for additional plant organellar RNA-editing studies to further clarify any phylogenetic trend. The position of hornworts in the land plant phylogeny remains ambiguous but pivotal, being either the earliest diverging land plants, sister to tracheophytes or sister to all other bryophytes [2]. Elucidating the evolutionary position of the hornworts will be essential to understand the polarity of RNA editing. If the hornworts are proven to be the sister group to all other land plants or part of a bryophyte clade, then our transcriptomic data for both the *L*. *dussii* plastome and mitogenome would indicate that reverse editing evolved independently in hornworts and seedless tracheophytes (lycopods, ferns). The alternative hypothesis would suggest that reverse editing arose in the most recent common ancestor of hornworts and tracheophytes, with subsequent secondary losses in some lycophytes, ferns and seed plants. In either scenario, hornworts remain a central group to unravel the genomic implications of RNA editing in land plants and its intriguing maintenance despite the evident evolutionary disadvantages.

## Supporting information

S1 Fig **A. Transcriptome read coverage of the *L*. *dussii* plastome.** The x-axis shows the gene map of this genome, starting with the LSC region, while the y-axis indicates read depth. **B**. **Transcriptome read coverage of the *L*. *dussii* mitogenome.** The x-axis shows the gene map of this genome, while the y-axis indicates read depth. A color-coded scale classifies the genes into functional categories.
(EPS)

S2 Fig Number of RNA edited sites per gene in the *Leiosporoceros* and *Anthoceros* plastomes. The numbers on the right of the histograms indicate the edited sites that are shared between the two hornworts.
(TIFF)

S3 Fig Percentage of reads in each edited site with respect to total reads in the plastome. Coverage goes from 165 times up to 65770 times (in position 85532, PsaM).
(PDF)

S4 Fig Percentage of reads in each edited site from total reads in the mitogenome. Coverage goes from 102 times up to 44015 times (in position 63551, nad5 gene).
(PDF)

S1 Table RNA edited sites in the plastome of *L*. *dussii*. (DOCX)

S2 Table RNA edited sites in the mitogenome of *L*. *dussii*. (DOCX)
