# Supplementary figures and images for: Genome-wide organellar analyses from the hornwort Leiosporoceros dussii show low frequency of RNA editing
Source: PLoS One. 2018 Aug 8;13(8):e0200491. doi: 10.1371/journal.pone.0200491 (PMC6082510; doi:10.1371/journal.pone.0200491)

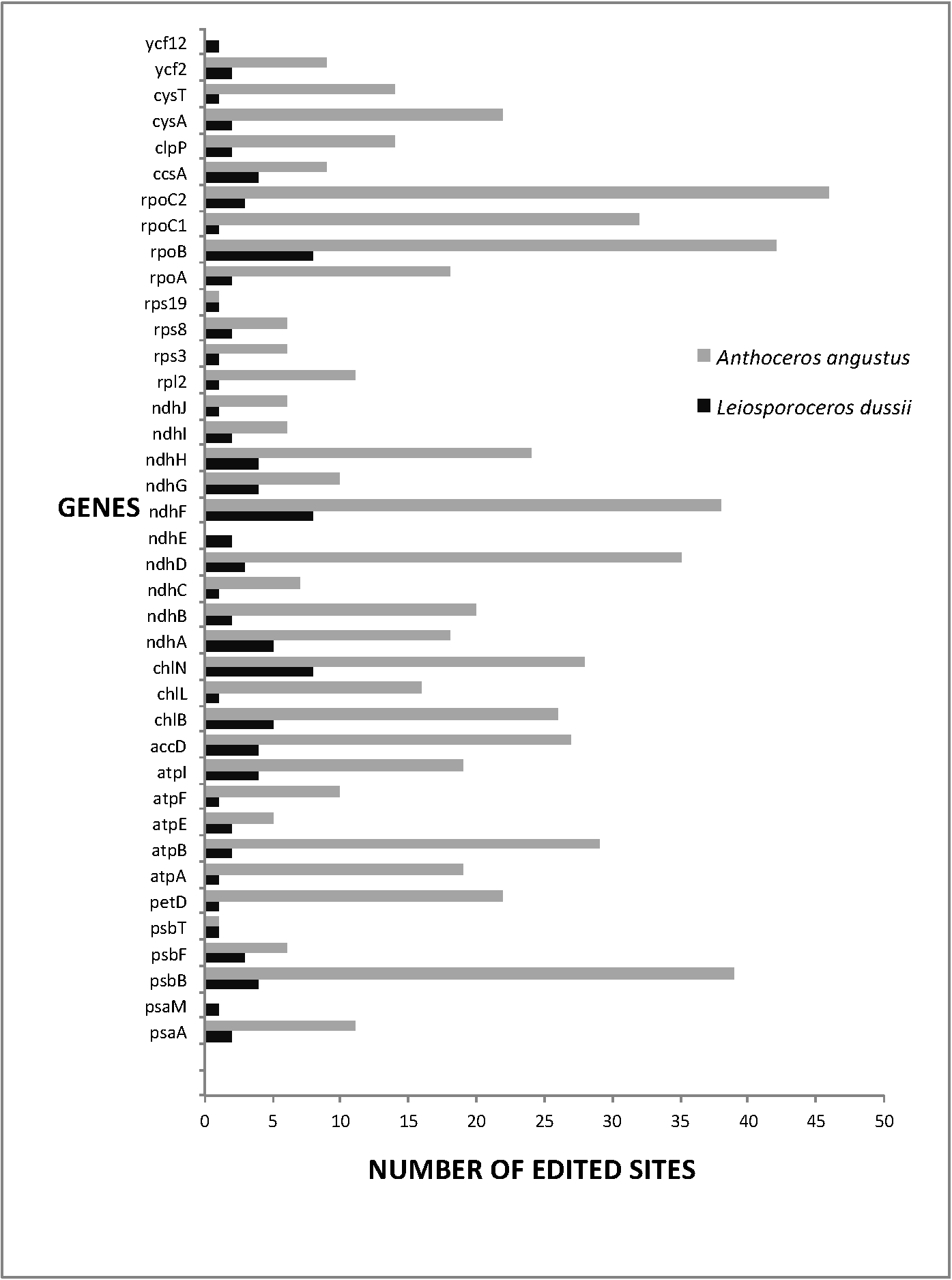

Supplement: S2 Fig — The numbers on the right of the histograms indicate the edited sites that are shared between the two hornworts. (TIFF) [file pone.0200491.s002.tiff]

Percentage of edited reads with respect to total read number

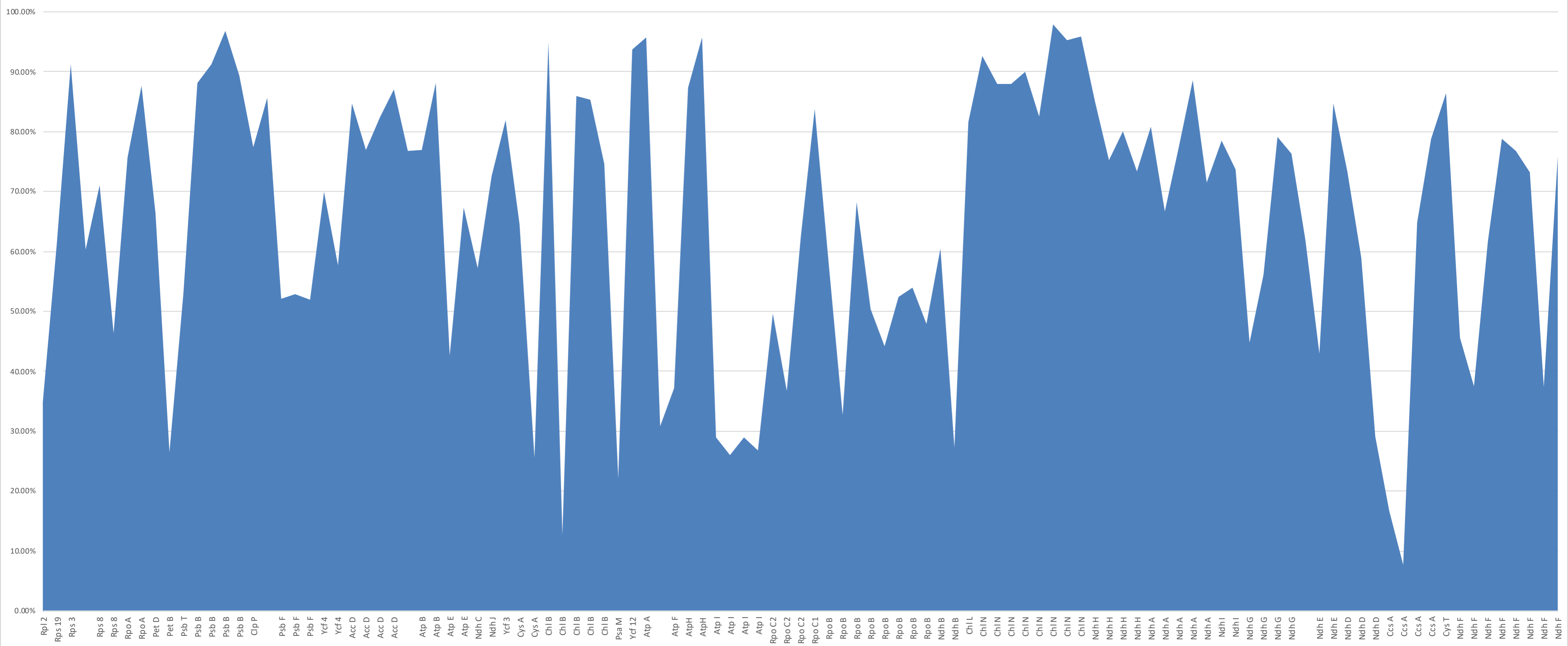

Supplement: S3 Fig — Coverage goes from 165 times up to 65770 times (in position 85532, PsaM). (PDF) [file pone.0200491.s003.pdf]

Percentage of reads edited with respect to total read number

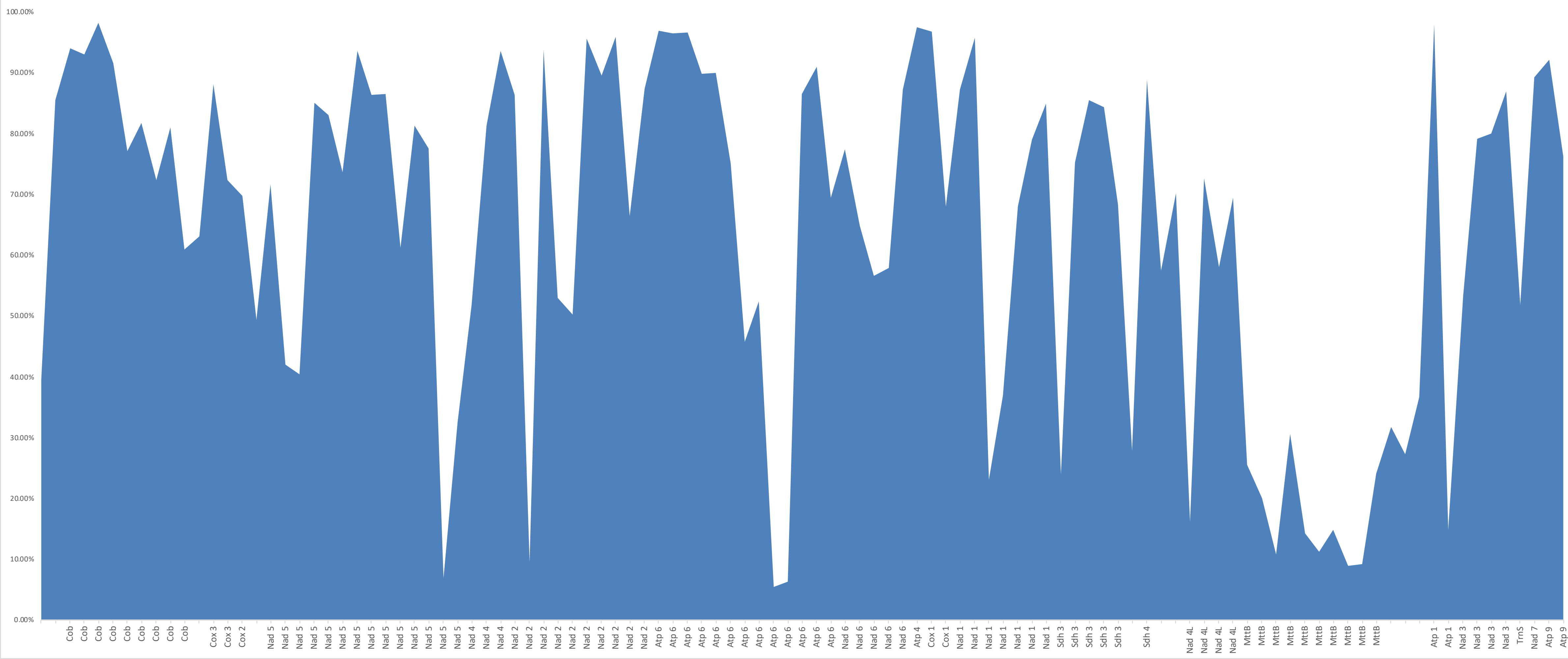

Supplement: S4 Fig — Coverage goes from 102 times up to 44015 times (in position 63551, nad5 gene). (PDF) [file pone.0200491.s004.pdf]
