# Supplementary material for: Genome-wide organellar analyses from the hornwort Leiosporoceros dussii show low frequency of RNA editing
Source: PLoS One. 2018 Aug 8;13(8):e0200491. doi: 10.1371/journal.pone.0200491 (PMC6082510; doi:10.1371/journal.pone.0200491)
Supplement: S1 Table — (DOCX) [file pone.0200491.s005.docx]

**Table S1. RNA edited sites in the plastome of *Leiosporoceros dussii.***

| Site of edition | # position | Intergenic region | Intron |  | Type of editing C-U, U-C | Codon position | Predicted codon | Edited codon |
| --- | --- | --- | --- | --- | --- | --- | --- | --- |
| 1 | 2724 |  |  | rpl2 | C-U | 2 | CCA: Pro | CUA: Leu |
| 2 | 4039 |  |  | rps19 | C-U | 2 | UCA: Ser | UUA: Leu |
| 3 | 5159 |  |  | rps3 | C-U | 2 | CCU: Pro | CUU: Leu |
| 4 | 8015 | rpl14-rps8 |  |  | C-U |  |  |  |
| 5 | 8158 |  |  | rps8 | C-U | 2 | UCA: Ser | UUA: Leu |
| 6 | 8449 |  |  | rps8 | C-U | 2 | UCA: Ser | UUA: Leu |
| 7 | 9917 |  |  | rpoA | C-U | 2 | UCG: Ser | UUG: Leu |
| 8 | 10571 |  |  | rpoA | C-U | 2 | UCA: Ser | UUA: Leu |
| 9 | 11200 |  |  | petD | C-U | 2 | CCA: Ser | UUG: Leu |
| 10 | 13868 |  | petB intron 1 | petB | C-U |  |  |  |
| 11 | 14999 |  |  | psbT | C-U | 2 | UCU: Ser | UUU: Phe |
| 12 | 15573 |  |  | psbB | C-U | 2 | GCU: Ala | GUU: Val |
| 13 | 15639 |  |  | psbB | C-U | 2 | UCG: Ser | UUG: Leu |
| 14 | 16125 |  |  | psbB | C-U | 2 | UCA: Ser | UUA: Leu |
| 15 | 16614 |  |  | psb B | C-U | 2 | UCU: Ser | UUU: Phe |
| 16 | 19102 |  |  | clpP | C-U | 2 | GCU: Ala | GUU: Val |
| 17 | 25648 | psbL-psbE |  |  | C-U |  |  |  |
| 18 | 26023 |  |  | psbF | C-U | 2 | UCA: Ser | UUA: Leu |
| 19 | 26032 |  |  | psbF | C-U | 2 | ACA: Thr | AUA: Ile |
| 20 | 26041 |  |  | psbF | C-U | 2 | ACG: Thr | AUG: Met |
| 21 | 30814 |  |  | ycf4 | C-U | 2 | UCG: Ser | UUG: Leu |
| 22 | 30862 |  |  | ycf4 | C-U | 2 | UCA: Ser | UUA: Leu |
| 23 | 32469 |  |  | accD | C-U | 2 | UCA: Ser | UUA: Leu |
| 24 | 32484 |  |  | accD | C-U | 2 | UCA: Ser | UUA: Leu |
| 25 | 32761 |  |  | accD | C-U | 1 | CGU: Arg | UGU: Cys |
| 26 | 33048 |  |  | accD | C-U | 2 | UCA: Ser | UUA: Leu |
| 27 | 36346 | rbcL-atpB |  |  | C-U |  |  |  |
| 28 | 36784 |  |  | atpB | C-U | 2 | UCU: Ser | UUU: Phe |
| 29 | 37441 |  |  | atpB | C-U | 2 | UCA: Ser | UUA: Leu |
| 30 | 37835 |  |  | atpE | C-U | 2 | CUA: Leu | UUA: Leu |
| 31 | 37899 |  |  | atpE | C-U | 2 | CCA: Pro | CUA: Leu |
| 32 | 40612 |  |  | ndhC | C-U | 2 | CCU: Pro | CUU: Leu |
| 33 | 42053 |  |  | ndhJ | C-U | 1 | CAA: Gln | UAA: Stop |
| 34 | 49228 |  | ycf 3 intron 2 | ycf 3 | C-U |  |  |  |
| 35 | 63801 |  |  | cysA | C-U | 2 | UCA: Ser | UUA: Leu |
| 36 | 63900 |  |  | cysA | C-U | 2 | ACG: Thr | AUG: Met |
| 37 | 81433 |  |  | chlB | C-U | 2 | ACA: Thr | AUA: Ile |
| 38 | 82079 |  |  | chlB | C-U | 1 | CUC: Leu | UUC: Phe |
| 39 | 82519 |  |  | chlB | C-U | 2 | CCU: Pro | CUU: Leu |
| 40 | 82609 |  |  | chlB | C-U | 2 | UCA: Ser | UUA: Leu |
| 41 | 82651 |  |  | chlB­ | C-U | 2 | ACU: Thr | AUU: Ile |
| 42 | 85532 |  |  | psaM | C-U | 3 | GCC: Ala | GCU: Ala |
| 43 | 86166 |  |  | ycf 12 | C-U | 2 | UCA: Ser | UUA: Leu |
| 44 | 88294 |  |  | atpA | C-U | 2 | ACA: Thr | AUA: Ile |
| 45 | 89331 | atpA-atpF |  |  | C-U |  |  |  |
| 46 | 90644 |  |  | atpF | C-U | 1 | CUU: Leu | UUU: Phe |
| 47 | 91130 |  |  | atpH | C-U | 2 | ACG: Thr | AUG: Met |
| 48 | 91274 |  |  | atpH | C-U | 2 | ACG: Thr | AUG: Start |
| 49 | 92155 |  |  | atpI | C-U | 2 | UCG: Ser | UUG: Leu |
| 50 | 92200 |  |  | atpI | C-U | 2 | CCU: Pro | CUU: Leu |
| 51 | 92260 |  |  | atpI | C-U | 2 | UCA: Ser | UUC: Phe |
| 52 | 92299 |  |  | atpI | C-U | 2 | CCU: Pro | CUU: Leu |
| 53 | 96550 |  |  | rpoC2 | C-U | 2 | UCU: Ser | UUU: Phe |
| 54 | 98245 |  |  | rpoC2 | C-U | 2 | CCG: Pro | CUG: Leu |
| 55 | 98419 |  |  | rpoC2 | C-U | 2 | CCU: Pro | CUU: Leu |
| 56 | 99510 |  |  | rpoC1 | C-U | 2 | UCU: Ser | UUU: Phe |
| 57 | 102582 |  |  | rpoB | C-U | 2 | UCA: Ser | UUA: Leu |
| 58 | 102789 |  |  | rpoB | C-U | 2 | UCG: Ser | UUC: Phe |
| 59 | 103590 |  |  | rpoB | C-U | 2 | UCG: Ser | UUG: Leu |
| 60 | 103746 |  |  | rpoB | C-U | 2 | UCA: Ser | UUA: Leu |
| 61 | 103767 |  |  | rpoB | C-U | 2 | UCA: Ser | UUA: Leu |
| 62 | 103863 |  |  | rpoB | C-U | 2 | UCG: Ser | UUG: Leu |
| 63 | 104196 |  |  | rpoB | C-U | 2 | UCA: Ser | UUA: Leu |
| 64 | 104505 |  |  | rpoB | C-U | 2 | UCA: Ser | UUA: Leu |
| 65 | 108351 |  |  | ndhB | C-U | 2 | CCU: Pro | CUU: Leu |
| 66 | 109588 |  |  | ndhB | C-U | 2 | UCU: Ser | UUU: Phe |
| 67 | 124444 |  |  | chlL | C-U | 2 | UCA: Ser | UUA: Leu |
| 68 | 124810 |  |  | chlN | C-U | 2 | UCU: Ser | UUU: Phe |
| 69 | 124897 |  |  | chlN | C-U | 2 | UCC: Ser | UUC: Phe |
| 70 | 124984 |  |  | chlN | C-U | 2 | UCA: Ser | UUA: Leu |
| 71 | 125002 |  |  | chlN | C-U | 2 | UCA: Ser | UUA: Leu |
| 72 | 125581 |  |  | chlN | C-U | 2 | ACU: Thr | AUU: Ile |
| 73 | 125767 |  |  | chlN | C-U | 2 | UCA: Ser | UUA: Leu |
| 74 | 125848 |  |  | chlN | C-U | 2 | UCA: Ser | UUA: Leu |
| 75 | 126067 |  |  | chlN | C-U | 2 | CCU: Pro | CUU: Leu |
| 76 | 132743 |  |  | ndhH | C-U | 2 | UCU: Ser | UUU: Phe |
| 77 | 132896 |  |  | ndhH | C-U | 2 | CCU: Pro | CUU: Leu |
| 78 | 133034 |  |  | ndhH | C-U | 2 | CCA: Pro | CUA: Leu |
| 79 | 133067 |  |  | ndhH | C-U | 2 | UCA: Ser | UUA: Leu |
| 80 | 133629 |  |  | ndhA | C-U | 2 | CCA: Pro | CUA: Leu |
| 81 | 133812 |  |  | ndhA | C-U | 2 | CCU: Pro | CUU: Leu |
| 82 | 134933 |  |  | ndhA | C-U | 2 | UCC: Ser | UUC: Phe |
| 83 | 134960 |  |  | ndhA | C-U | 2 | UCA: Ser | UUA: Leu |
| 84 | 135062 |  |  | ndhA | C-U | 2 | CCU: Pro | CUU: Leu |
| 85 | 135852 |  |  | ndhI | C-U | 2 | CCU: Pro | CUU: Leu |
| 86 | 135876 |  |  | ndhI | C-U | 2 | UCA: Ser | UUA: Leu |
| 87 | 136411 |  |  | ndhG | C-U | 2 | UCG: Ser | UUG: Leu |
| 88 | 136528 |  |  | ndhG | C-U | 2 | GCU: Ala | GUU: Val |
| 89 | 136771 |  |  | ndhG | C-U | 2 | CCU: Pro | CUU: Leu |
| 90 | 136786 |  |  | ndhG | C-U | 2 | UCA: Ser | UUA: Leu |
| 91 | 136959 | ndhG- ndhE |  |  | C-U | 2 |  |  |
| 92 | 136979 |  |  | ndhE | C-U | 2 | CCU: Pro | CUU: Leu |
| 93 | 137153 |  |  | ndhE | C-U | 2 | UCU: Ser | UUU: Phe |
| 94 | 138503 |  |  | ndhD | C-U | 2 | UCA: Ser | UUA: Leu |
| 95 | 138635 |  |  | ndhD | C-U | 2 | UCA: Ser | UUA: Leu |
| 96 | 139498 |  |  | ndhD | C-U | 1 | CGU: Arg | UGU: Cys |
| 97 | 139968 |  |  | ccsA | C-U | 2 | UCU: Ser | UUU: Phe |
| 98 | 139971 |  |  | ccsA | C-U | 2 | UCU: Ser | UUU: Phe |
| 99 | 140136 |  |  | ccsA | C-U | 2 | CCU: Pro | CUU: Leu |
| 100 | 140493 |  |  | ccsA | C-U | 2 | UCA: Ser | UUA: Leu |
| 101 | 141817 |  |  | cysT | C-U | 2 | UCA: Ser | UUA: Leu |
| 102 | 143998 |  |  | ndhF | C-U | 2 | CCC: Pro | CUC: Leu |
| 103 | 144322 |  |  | ndhF | C-U | 2 | ACG: Thr | AUG: Met |
| 104 | 144499 |  |  | ndhF | C-U | 2 | UCA: Ser | UUA: Leu |
| 105 | 144745 |  |  | ndhF | C-U | 2 | CCU: Pro | CUU: Leu |
| 106 | 144826 |  |  | ndhF | C-U | 2 | UCA: Ser | UUA: Leu |
| 107 | 145126 |  |  | ndhF | C-U | 2 | UCU: Ser | UUU: Phe |
| 108 | 145240 |  |  | ndhF | C-U | 2 | UCA: Ser | UUA: Leu |
| 109 | 145549 |  |  | ndhF | C-U | 2 | CCA: Pro | CUA: Leu |
| Total | 109 | 5 | 2 |  |  |  |  |  |
