# Supplementary material for: Genome-wide organellar analyses from the hornwort Leiosporoceros dussii show low frequency of RNA editing
Source: PLoS One. 2018 Aug 8;13(8):e0200491. doi: 10.1371/journal.pone.0200491 (PMC6082510; doi:10.1371/journal.pone.0200491)
Supplement: S2 Table — (DOCX) [file pone.0200491.s006.docx]

**Table S2. RNA edited sites in the mitogenome of *Leiosporoceros dussii*.**

| **Site of edition** | **# position** | **Intergenic region** | **Intron** |  | **Type of editing C-U, U-C, other** | **Codon position** | **Predicted codon** | **Edited codon** |
| --- | --- | --- | --- | --- | --- | --- | --- | --- |
| 1 | 2483 | trnMF-rns |  |  | C-U |  |  | CCA: Pro |
| 2 | 8338 | trnMe-rrn5 |  |  | U-C |  |  |  |
| 3 | 18228 |  |  | cob | C-U | 2 | UCU: Ser | UUU: Phe |
| 4 | 18242 |  | ­­­­ | cob | C-U | 3 | AUC: Ile | AUU: Ile |
| 5 | 18408 |  |  | cob | C-U | 2 | UCC: Ser | UUC: Phe |
| 6 | 18414 |  |  | cob | C-U | 2 | CCA: Pro | CUA: Leu |
| 7 | 26040 |  |  | cob | C-U | 2 | UCA: Ser | UUA: Leu |
| 8 | 26106 |  |  | cob | C-U | 2 | UCA: Ser | UUA: Leu |
| 9 | 26281 |  |  | cob | U-C | 1 | UAG: Stop | CAG: Gln |
| 10 | 26289 |  |  | cob | C-U | 2 | UCG: Ser | UUG: Leu |
| 11 | 26415 |  |  | cob | C-U | 2 | ACG: Thr | AUG: Met- start |
| 12 | 26419 | cob-cox3 |  |  | C-U |  |  |  |
| 13 | 27032 |  |  | cox3 | U-C | 2 | UUC: Phe | UCC: Ser |
| 14 | 30284 |  |  | cox3 | U-C | 2 | CUA: Leu | CCU: Pro |
| 15 | 30381 |  |  | cox3 | C-U | 1 | CGG: Arg | UGG: Trp |
| 16 | 52498 | Rpl10-trnl(caa) |  |  | U-C |  |  |  |
| 17 | 54908 |  |  | nad5 | U-C | 1 | UGA: Stop | CGA: Arg |
| 18 | 55052 |  |  | nad5 | C-U | 1 | CGG: Arg | UGG: Trp |
| 19 | 55907 |  | nad5 intron 1 | nad5 | C-U |  |  |  |
| 20 | 56120 |  |  | nad5 | U-C | 2 | UUA: Leu | UCA: Ser |
| 21 | 56183 |  |  | nad5 | C-U | 2 | ACG: Thr | AUG: Met |
| 22 | 56194 |  |  | nad5 | U-C | 1 | UGA: Stop | CGA: Arg |
| 23 | 56353 |  |  | nad5 | C-U | 1 | CGU: Arg | UGU: Cys |
| 24 | 56363 |  |  | nad5 | C-U | 2 | CCU: Pro | CUU: Leu |
| 25 | 56489 |  |  | nad5 | C-U | 2 | UCU: Ser | UUU: Phe |
| 26 | 56516 |  |  | nad5 | C-U | 2 | UCC: Ser | UUC: Phe |
| 27 | 63550 |  |  | nad5 | C-U | 2 | UCC: Ser | UUC: Phe |
| 28 | 63551 |  |  | nad5 | C-U | 3 | UCC: Ser | UCU: Ser |
| 29 | 63614 |  |  | nad5 | C-U | 3 | CUC: Leu | CUU: Leu |
| 30 | 63627 |  |  | nad5 | U-C | 1 | UGA: Stop | CGA: Arg |
| 31 | 66734 |  |  | nad4 | C-U | 2 | ACG: Thr | AUG: Met- start |
| 32 | 67133 |  |  | nad4 | C-U | 2 | UCU: Ser | UUU: Phe |
| 33 | 67499 |  |  | nad4 | C-U | 2 | CCU: Pro | CUU: Leu |
| 34 | 71307 |  |  | nad2 | C-U | 2 | CCU: Pro | CUU: Leu |
| 35 | 71365 |  |  | nad2 | C-U | 3 | UUC: Phe | UUU: Phe |
| 36 | 71508 |  |  | nad2 | C-U | 2 | CCU: Pro | CUU: Leu |
| 37 | 71774 |  |  | nad2 | C-U | 1 | CUG: Leu | UUG: Leu |
| 38 | 71786 |  |  | nad2 | U-C | 1 | UAA: Stop | CAA: Gln |
| 39 | 73615 |  |  | nad2 | C-U | 2 | CCA: Pro | CUA: Leu |
| 40 | 73684 |  |  | nad2 | C-U | 2 | UCA: Ser | UUA: Leu |
| 41 | 73807 |  |  | nad2 | C-U | 2 | ACG: Thr | AUG: Met |
| 42 | 73868 |  |  | nad2 | C-U | 3 | UUC: Phe | UUU: Phe |
| 43 | 76274 |  |  | nad2 | C-U | 2 | UCU: Ser | UUU: Phe |
| 44 | 81247 |  |  | atp6 | C-U | 2 | ACU: Thr | AUU: Ile |
| 45 | 81268 |  |  | atp6 | C-U | 2 | UCA: Ser | UUA: Leu |
| 46 | 81368 |  |  | atp6 | C-U | 1 | CGG: Arg | UGG: Trp |
| 47 | 81458 |  |  | atp6 | C-U | 1 | CAU: His | UAU: Tyr |
| 48 | 81461 |  |  | atp6 | C-U | 1 | CCU: Pro | UCU: Ser |
| 49 | 81528 |  | atp6 intron2 | atp6 | C-U |  |  |  |
| 50 | 84712 |  |  | atp6 | C-U | 2 | CCU: Pro | CUU: Leu |
| 51 | 84718 |  |  | atp6 | C-U | 2 | CCU: Pro | CUU: Leu |
| 52 | 84773 |  |  | atp6 | C-U | 1 | CAU: His | UAU: Tyr |
| 53 | 84775 |  |  | atp6 | U-C | 2 | CUU: Leu | CCU: Pro |
| 54 | 84797 |  |  | atp6 | C-U | 1 | CGU: Arg | UGU: Cys |
| 55 | 84809 |  |  | atp6 | C-U | 1 | CGU: Arg | UGU: Cys |
| 56 | 85725 |  |  | atp6 | C-U | 2 | UCG: Ser | AUG: Met |
| 57 | 89210 |  |  | nad6 | C-U | 1 | CGC: Arg | UGC: Cys |
| 58 | 89276 |  |  | nad6 | C-U | 1 | CAU: His | UAU: Tyr |
| 59 | 89301 |  |  | nad6 | C-U | 2 | UCU: Ser | UUU: Phe |
| 60 | 89304 |  |  | nad6 | C-U | 2 | CCA: Pro | CUA: Leu |
| 61 | 89391 |  |  | nad6 | C-U | 2 | CCU: Pro | CUU: Leu |
| 62 | 95445 |  |  | atp4 | C-U | 2 | CCG: Pro | CUG: Leu |
| 63 | 101459 |  |  | cox1 | C-U | 2 | UCA: Ser | UUA: Leu |
| 64 | 104500 |  |  | cox1 | C-U | 3 | CUC: Leu | CUU: Leu |
| 65 | 111484 |  |  | nad1 | C-U | 2 | UCA: Ser | UUA: Leu |
| 66 | 111511 |  |  | nad1 | C-U | 2 | ACG: Thr | AUG: Met |
| 67 | 113118 |  |  | nad1 | U-C | 2 | UUU: Phe | UCU: Ser |
| 68 | 113144 |  |  | nad1 | U-C | 2 | UGA: Stop | CGA: Arg |
| 69 | 113222 |  |  | nad1 | U-C | 1 | CGC: Arg | UGC: Cys |
| 70 | 113249 |  |  | nad1 | U-C | 1 | UAA: Stop | CAA: Gln |
| 71 | 113309 |  |  | nad1 | C-U | 1 | CGU: Arg | UGU: Cys |
| 72 | 135913 |  |  | sdh3 | C-U | 1 | CUU: Leu | UUU: Phe |
| 73 | 136033 |  |  | sdh3 | C-U | 1 | CGC: Arg | UGC: Cys |
| 74 | 136064 |  |  | sdh3 | C-U | 2 | UCA: Ser | UUA: Leu |
| 75 | 136076 |  |  | sdh3 | C-U | 2 | CCG: Pro | CUG: Leu |
| 76 | 136165 |  |  | sdh3 | U-C | 1 | UGA: Stop | CGA: Arg |
| 77 | 136226 | sdh3- sdh4 |  |  | C-U |  |  |  |
| 78 | 136887 |  |  | sdh4 | C-U | 2 | ACU: Thr | AUU: Ile |
| 79 | 137416 | sdh4- nad4L |  |  | C-U |  |  |  |
| 80 | 137531 | sdh4- nad4Ls |  |  | C-U |  |  |  |
| 81 | 138056 |  |  | nad4L | C-U | 2 | CCU: Pro | CUU: Leu |
| 82 | 138071 |  |  | nad4L | U-C | 2 | UUG: Leu | UCG: Ser |
| 83 | 138092 |  |  | nad4L | U-C | 2 | GUG: Val | GCG: Ala |
| 84 | 138251 |  |  | nad4L | C-U | 2 | UCU: Ser | UUU: Phe |
| 85 | 139479 |  |  | mttB | C-U | 1 | CGG: Arg | UGG: Trp |
| 86 | 139491 |  |  | mttB | C-U | 1 | CGC: Arg | UGC: Cys |
| 87 | 139544 |  |  | mttB | C-U | 3 | UUC: Phe | UUU: Phe |
| 88 | 139564 |  |  | mttB | C-U | 2 | CCU: Pro | CUU: Leu |
| 89 | 139618 |  |  | mttB | C-U | 2 | CCG: Pro | CUG: Leu |
| 90 | 139650 |  |  | mttB | C-U | 1 | CGC: Arg | UGC: Cys |
| 91 | 140036 |  |  | mttB | C-U | 3 | UUC: Phe | UUU: Phe |
| 92 | 140101 |  |  | mttB | C-U | 2 | UCU: Ser | UUU: Phe |
| 93 | 140113 |  |  | mttB | C-U | 2 | UCG: Ser | UUG: Leu |
| 94 | 140122 |  |  | mttB | C-U | 2 | UCU: Ser | UUU: Phe |
| 95 | 140185 | mttB-rps4 |  |  | C-U |  |  |  |
| 96 | 140190 | mttB-rps4 |  |  | C-U |  |  |  |
| 97 | 140241 | mttB-rps4 |  |  | C-U |  |  |  |
| 98 | 164235 |  |  | atp1 | C-U | 2 | CCG: Pro | CUG: Leu |
| 99 | 171390 |  |  | atp1 | C-U | 1 | CUA: Leu | UUA: Leu |
| 100 | 175229 |  |  | nad3 | C-U | 1 | CAC: His | UAC: Tyr |
| 101 | 178073 |  |  | nad3 | C-U | 2 | UCU: Ser | UUU: Phe |
| 102 | 178076 |  |  | nad3 | C-U | 2 | UCA: Ser | UUA: Leu |
| 103 | 178151 |  |  | nad3 | C-U | 2 | UCG: Ser | UUG: Leu |
| 104 | 183147 |  |  | trnS(gcu) | C-U |  |  |  |
| 105 | 186639 |  |  | nad7 | U-C |  |  |  |
| 106 | 204127 |  |  | atp9 | C-U | 2 | UCA: Ser | UUA: Leu |
| 107 | 209483 |  |  | atp9 | C-U | 2 | UCA: Ser | UUA: Leu |
| 108 | 22668 |  |  | Orf669 | U-C | 1 | TGA: stop | CGA: Arg |
| Total | 108 | 10 | 2 |  |  |  |  |  |
